# Persistently normal blood tests in patients taking methotrexate for RA or azathioprine for IBD: a retrospective cohort study

**DOI:** 10.3399/BJGP.2021.0595

**Published:** 2022-03-08

**Authors:** Simon DS Fraser, Sharon X Lin, Matthew Stammers, David Culliford, Kinda Ibrahim, Ravina Barrett, Clare Howard, Ruth Johnson, Nicola Barnes, James Batchelor, Christopher Holroyd, Jo Adams, Adam Rischin, Paul Roderick, Paul Rutter, Christopher J Edwards

**Affiliations:** School of Primary Care, Population Science and Medical Education, Faculty of Medicine, University of Southampton, Southampton, UK.; Southampton General Hospital, Southampton, UK.; University Hospital Southampton NHS Foundation Trust, Southampton, UK.; National Institute for Health Research Applied Research Collaboration Wessex, School of Health Sciences, University of Southampton, Southampton, UK.; Academic Geriatric Medicine, Faculty of Medicine, University of Southampton, Southampton, UK.; School of Pharmacy and Biomolecular Sciences, University of Brighton, Brighton, UK.; Wessex Academic Health Science Network, Chilworth, UK.; Commissioning Support Unit, NHS South, Central and West, Eastleigh, UK.; School of Pharmacy and Biomedical Sciences, University of Portsmouth, Portsmouth, UK.; Clinical Informatics Research Unit, Faculty of Medicine, University of Southampton, Southampton, UK.; University Hospital Southampton NHS Foundation Trust, Southampton, UK.; School of Health Sciences, University of Southampton, Southampton, UK.; Alfred Health, Melbourne, Australia.; School of Primary Care, Population Science and Medical Education, Faculty of Medicine, University of Southampton, Southampton, UK.; School of Pharmacy and Biomedical Sciences, University of Portsmouth, Portsmouth, UK.; Clinical Rheumatology, University of Southampton, Southampton, UK.

**Keywords:** antirheumatic agents, inflammatory bowel diseases, rheumatoid arthritis

## Abstract

**Background:**

Disease-modifying anti-rheumatic drugs (DMARDs), including methotrexate and azathioprine, are commonly used to treat rheumatoid arthritis (RA) and inflammatory bowel disease (IBD). Blood-test safety monitoring is mainly undertaken in primary care. Normal blood results are common.

**Aim:**

To determine the frequency and associations of persistently normal blood tests in patients with RA prescribed methotrexate, and patients with IBD prescribed azathioprine.

**Design and setting:**

Two-year retrospective study of a cohort taken from an electronic pseudonymised primary care/laboratory database covering >1.4 million patients across Hampshire, UK.

**Method:**

Patients with RA and IBD, and associated methotrexate and azathioprine prescriptions, respectively, were identified. Tests and test thresholds recommended by the National Institute for Health and Care Excellence were applied. Persistent normality was defined as no abnormalities of any tests nor alanine aminotransferase (ALT), white blood count (WBC), neutrophils, and estimated glomerular filtration rate (eGFR) individually. Logistic regression was used to identify associations with test normality.

**Results:**

Of 702 265 adults, 7102 had RA and 8597 had IBD. In total, 3001 (42.3%) patients with RA were prescribed methotrexate and 1162 (13.5%) patients with IBD were prescribed azathioprine; persistently normal tests occurred in 1585 (52.8%) and 657 (56.5%) of the populations, respectively. In patients with RA on methotrexate, 585 (19.5%) had eGFR, 219 (7.3%) ALT, 217 (7.2%) WBC, and 202 (6.7%) neutrophil abnormalities. In patients with IBD on azathioprine, 138 (11.9%) had WBC, 88 (7.6%) eGFR, 72 (6.2%) ALT, and 65 (5.6%) neutrophil abnormalities. Those least likely to have persistent test normality were older and/or had comorbidities.

**Conclusion:**

Persistent test normality is common when monitoring these DMARDs, with few hepatic or haematological abnormalities. More stratified monitoring approaches should be explored.

## INTRODUCTION

Rheumatoid arthritis (RA) and inflammatory bowel disease (IBD) are common inflammatory conditions, with a prevalence of 0.8% (two to three times higher among women than men) and 0.7% (ulcerative colitis approximately 0.4%, Crohn’s disease 0.3%), respectively.^1^^,^^2^ Disease-modifying anti-rheumatic drugs (DMARDs), including synthetic and biological agents, are used to control disease activity and progression.^3^^–^6 The most common synthetic DMARDs in the UK are methotrexate for RA and azathioprine for IBD.^3^^–^5 Both require regular safety blood-test monitoring for liver-function abnormalities and kidney-function and bone-marrow toxicity; this is undertaken frequently at initiation, then less frequently once a maintenance dose is established.^7^ In the UK, methotrexate or azathioprine initiation usually occurs in secondary care, with blood-test monitoring and ongoing repeat prescribing undertaken in primary care, as recommended by the National Institute for Health and Care Excellence (NICE).^7^^,^^8^ Guidelines include recommendations to *‘consider stopping treatment and referring urgently’* if results show abnormalities.^7^^,^^9^

Regular blood-test monitoring has been linked with anxiety and depression for some patients; it also incurs substantial costs for healthcare providers, and increases the workload for clinicians, laboratory staff, and administrators.^10^^–^13 Despite guidelines, the optimal monitoring frequency has not been established^3^^–^6 and the extent to which patients taking DMARDs experience prolonged periods with no abnormal tests is unclear. A review of RA monitoring schedules concluded that drug-related toxicities are infrequent and that clear evidence for monitoring preventing harm is lacking.^14^ Targeted monitoring of higher-risk individuals and reduced monitoring for lower-risk patients may improve health-service efficiency and reduce patient workload, provided safety is established.

**Table:** How this fits in

Clinical guidance from the National Institute for Health and Care Excellence recommends 3-monthly blood-tests for the ongoing safety monitoring of conventional synthetic disease-modifying anti-rheumatic drugs, but questions have been raised about the need for this testing frequency. Using 2 years’ data from a large primary care database, this study found that persistent normality of blood-test results was common and abnormalities were dominated by reduced renal function among older people, with relatively few hepatic or haematological abnormalities. Greater stratification of monitoring may reduce workload and costs for patients and health services, but more evidence is required on the long-term safety, acceptability, and cost-effectiveness of changing current practice.

In order to inform monitoring strategies, this study aimed to assess the extent of, and factors associated with, persistently normal NICE-recommended blood-test results among people with RA taking methotrexate and patients with IBD taking azathioprine. It also aimed to describe the frequency of blood testing to give an indication of health-service and patient workload.

## METHOD

The study cohort was taken from the Care and Health Information Analytics (CHIA) database; this pseudonymised electronic database contains primary care data for >1.4 million patients registered with GP surgeries across Hampshire and the Isle of Wight, UK, with linked clinical biochemistry and haematology data from two large NHS hospital laboratories (Southampton and Portsmouth). The study population consisted of adults (aged ≥18 years) who were:

- registered with GP practices that consistently sent all laboratory data to one of the two hospitals for the entire study duration; and
- alive on 1 October 2017 and survived until 30 September 2019.

It was, therefore, a retrospective ‘closed’ cohort, for whom clinical diagnoses and complete biochemical and haematological data were available (both GP-ordered tests and all hospital-ordered tests).^15^ ‘Baseline’ was defined as the first 2-month study period (October 2017–November 2017) to describe prescribing patterns.

RA and IBD were identified using Read codes and described as:

- prevalent — a diagnosis of RA or IBD was in the medical record at baseline; or
- incident — the first RA or IBD coding occurred during follow-up (October 2017–September 2019).

DMARD prescription was defined as having at least one primary care-issued prescription of methotrexate or azathioprine in any 2-month period across the 2 years. Periods of 2 months were chosen because repeat prescribing in the UK commonly adopts a 1-month or 2-month prescription pattern. Finer data cuts might mislabel some as being prescribed or not prescribed at any given time.

Age was defined in years, based on birth year prior to pseudonymisation (date of birth was not available to the study team). Ethnicity was categorised into: British and mixed British/Irish/other White; mixed (White and Asian, White and Black African, White and Black Caribbean, and other mixed); Indian/Bangladeshi/Pakistani/other Asian; African/Caribbean/other Black; and other. Socioeconomic status was defined using 2015 Index of Multiple Deprivation (IMD) quintiles.^16^ The IMD is a small-area measure of socioeconomic status based on postcode, ranked nationally, comprising seven domains: income, employment, education/skills/training, health and disability, crime, barriers to housing and services, and living environment. Comorbidities were defined using Read codes, as described in a previous study;^17^ not all conditions were available in the dataset. Blood-test data included liver function, renal function, and full blood count.

### Outcomes

The main outcomes were lack of blood test abnormality while being prescribed methotrexate for RA or azathioprine for IBD. People in both disease/DMARD groups (that is, the RA/methotrexate and IBD/azathioprine groups) were identified who met all of the normality thresholds specified by NICE, namely:

- white blood count (WBC): ≥3.5×10^9^/L;
- mean cell volume: ≤105 fL;
- neutrophils: ≥1.6×10^9^/L;
- platelets: ≥140×10^9^/L;
- eosinophils: ≤0.5×10^9^/L;
- alanine aminotransferase (ALT): ≤100 U/L;
- aspartate transaminase (AST): ≤100 U/L;
- albumin: ≥30 g/L;
- estimated glomerular filtration rate (eGFR): ≥60 ml/min/1.73 m.^2^^,^^7^

ALT, eGFR, WBC, and neutrophils were then considered individually, using the NICE-recommended thresholds. NICE’s monitoring regime is detailed in Supplementary Box S1.^7^^,^^8^

### Statistical analyses

Descriptive statistics were used to identify prevalence and patterns of methotrexate and azathioprine exposure in each 2-month period among people with RA and IBD at baseline, and people with incident RA and IBD across the study period.

Total numbers of ALT, eGFR, and WBC tests were identified for the entire RA and IBD populations, those with RA taking methotrexate, those with IBD taking azathioprine, and for those with RA and IBD taking methotrexate and azathioprine continually over 2 years. To explore undermonitoring, the proportions of those with RA and IBD taking methotrexate and azathioprine continually over 2 years, with tests in all eight 3-month testing periods — then in seven periods, then six, five, and so on — was calculated for ALT, WBC, and eGFR tests.

Sociodemographic characteristics of those with ≤6 ALT, eGFR, and WBC tests were identified. Given the eight 3-month periods in the 24 months covered by this study, and the potential for individuals to have had a blood test just before or just after the study period, ≤6 was chosen as a way of reflecting failure to adhere to the recommended 3-monthly pattern.

Incidence of normal and abnormal blood tests was established using the stated NICE-specified tests and thresholds. The numbers and characteristics of people with normality of all tests were identified and described for people with RA and IBD for any period of methotrexate and azathioprine prescription, respectively. Numbers/proportions of people with persistent normality of individual tests (ALT, eGFR, WBC, and neutrophils) were described.

Multivariable logistic regression was used to explore associations between patient characteristics and persistent NICE blood test normality in the RA/methotrexate and IBD/azathioprine populations.

Absolute risk of blood-test normality was calculated for specific age and comorbidity groups for each condition, using NICE thresholds and tests; red/amber/green risk diagrams were developed using exploratory thresholds to demonstrate the potential utility of such risk stratification in clinical practice.

All analyses were conducted in R (version 3.6.3).^18^ The study is reported using The REporting of studies Conducted using Observational Routinely-collected health Data (RECORD) Statement (see Supplementary Table S1).^19^ Data extraction from the CHIA database was approved by the Care and Health Information Exchange Information Governance Group.

## RESULTS

**Figure 1.**
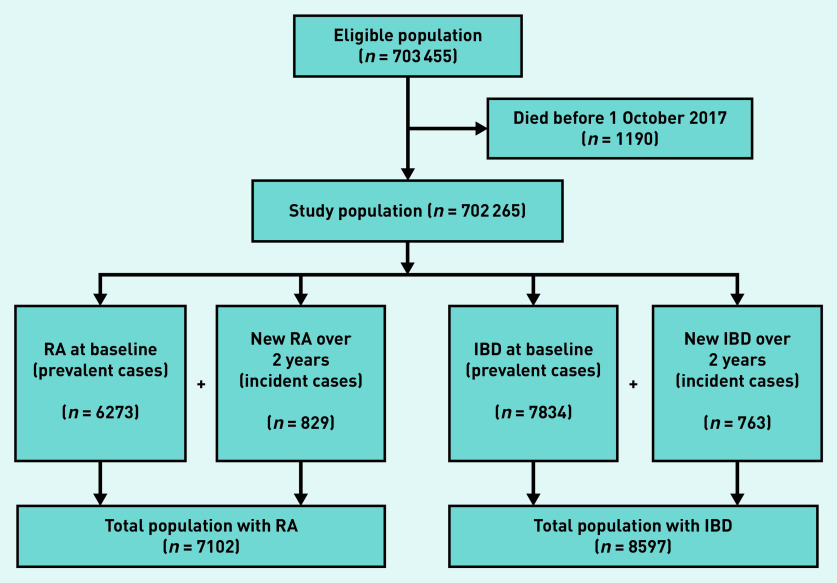
*Flowchart of study participant selection. IBD = inflammatory bowel disease. RA = rheumatoid arthritis.*

The cohort comprised 702 265 people registered with 85 GP practices; baseline characteristics are outlined in Table 1. The mean age was 51.9 years, 53.2% were female, and 14.6% lived in areas of greatest deprivation. Most were of British and mixed British/Irish/other White ethnicity, although ethnicity data were missing for 35.0% of the cohort. At baseline, 6273 (1.0%) had RA and 7834 (1.1%) IBD. The study process is outlined in Figure 1; during the 2-year study period, an additional 829 people and 763 people were diagnosed with RA and IBD, respectively.

**Table 1.** Cohort characteristics at baseline, *N* = 702 265

**Characteristic**	***n* (%)^a^**
**Age, years**	
Median (range)	52 (18–110)
Mean (SD)	51.9 (18.8)

**Age group, years**	
18–39	210 047 (29.9)
40–59	241 831 (34.4)
60–79	194 168 (27.6)
≥80	56 219 (8.0)

**Sex**	
Female	373 553 (53.2)
Male	328 712 (46.8)

**Socioeconomic status, IMD quintile**	
1 (most deprived)	102 796 (14.6)
2	126 069 (18.0)
3	132 262 (18.8)
4	146 622 (20.9)
5 (least deprived)	186 284 (26.5)
Missing	8232 (1.2)

**Ethnicity**	
British and mixed British/Irish/other White	427 019 (60.8)
Mixed (White and Asian, White and Black African, White and Black Caribbean, and other mixed)	3863 (0.6)

Indian/Bangladeshi/Pakistani/other Asian	13 180 (1.9)
African/Caribbean/other Black	5251 (0.7)
Other	7236 (1.0)
Missing	245 716 (35.0)

**Long-term conditions at baseline**	
Rheumatoid arthritis	6723 (1.0)
Inflammatory bowel disease	7834 (1.1)
Hypertension	150 458 (21.4)
Osteoarthritis	95 294 (13.6)
Diabetes	62 767 (8.9)
Cardiovascular disease^b^	52 985 (7.5)
Chronic kidney disease	36 731 (5.2)
Heart failure (including cor pulmonale)	12 799 (1.8)

**Prescribed at baseline**	
Methotrexate	2214 (0.3)
Azathioprine	746 (0.1)
Non-steroidal anti-inflammatory drug	19 364 (2.8)
Diuretic	35 912 (5.1)
Angiotensin-converting enzyme inhibitor	55 606 (7.9)
Angiotensin-II receptor antagonist	25 345 (3.6)

a *Unless otherwise stated.*

b *Includes ischaemic heart disease, cerebrovascular disease, and peripheral vascular disease. IMD = Index of Multiple Deprivation. SD = standard deviation.*

At baseline, 2214 (35.3%) people with RA were prescribed methotrexate and 746 (9.5%) people with IBD were prescribed azathioprine. The total number of people with RA (prevalent and incident) was 7102, of whom 3001 (42.3%) were prescribed methotrexate over 2 years. There were 8597 people with IBD (prevalent and incident), of whom 1162 (13.5%) were prescribed azathioprine. The overall percentage prescribed fell slightly for both drugs over the 2 years in this closed cohort, with only small numbers of people with IBD prescribed methotrexate and even smaller numbers with RA prescribed azathioprine (see Supplementary Figure S1 and Supplementary Box S2).

There were 44 838 and 12 724 ALT tests in the full RA and IBD populations, respectively; 47 772 (RA) and 13 739 (IBD) WBC tests; and 95 162 (RA) and 26 673 (IBD) eGFR tests. Both populations showed considerable variation in testing frequency, with eGFR tests being undertaken most frequently and most people having ≥9 blood tests over 2 years (see Supplementary Figure S2).

**Figure 2.**
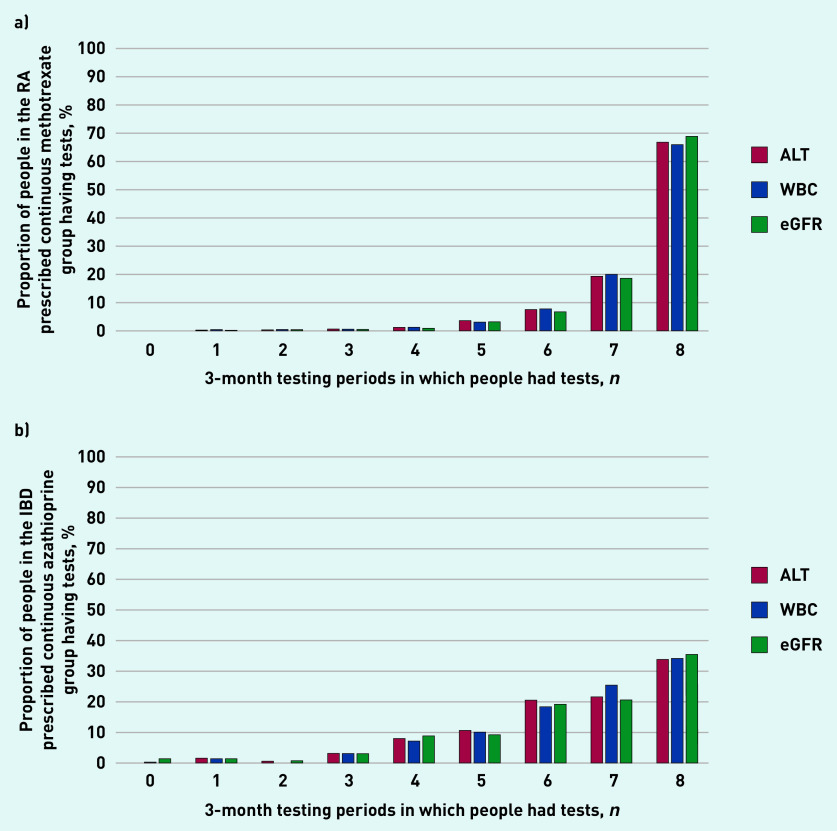
*Study participants who had blood tests, by number of 3-month testing periods (total study period = 24 months). ALT = alanine aminotransferase. eGFR = estimated glomerular filtration rate. IBD = inflammatory bowel disease. RA = rheumatoid arthritis. WBC = white blood count.*

Among the 1361 people with RA at baseline who were prescribed 2 years’ methotrexate continually, the majority (>65%) had ALT, WBC, and eGFR testing in every 3-month period (Figure 2a). In contrast, among 365 people with IBD at baseline who were prescribed azathioprine continually, <40% had testing in every 3-month period (Figure 2b).

The sociodemographic characteristics of those having ≤6 tests were similar to those of the full RA/methotrexate and IBD/azathioprine populations (see Supplementary Table S2).

### RA/methotrexate cohort

**Figure 3.**
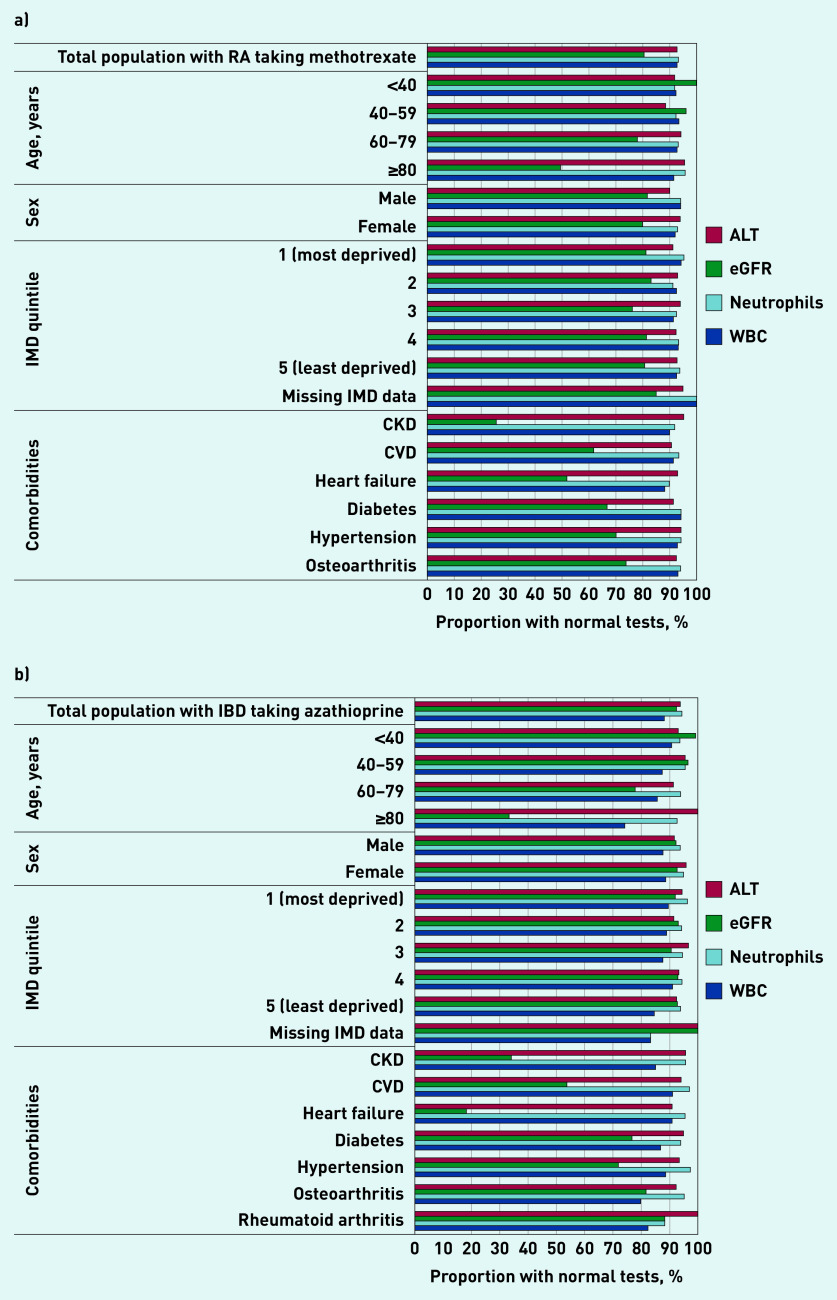
***Proportions of patients with normal blood-test results for specific tests by exposure categories. Test thresholds: WBC*** ≥***3.5×10*** *^9^****/L; MCV*** ≤***105 fL; neutrophils*** ≥***1.6×10*** *^9^****/L; ALT*** ≤***100 U/L; AST*** ≤***100 U/L; eGFR*** ≥***60 ml/min/1.73 m^2^. ALT = alanine aminotransferase. AST = aspartate transaminase. CKD = chronic kidney disease. CVD = cardiovascular disease. eGFR = estimated glomerular filtration rate. IBD = inflammatory bowel disease. IMD = Index of Multiple Deprivation. RA = rheumatoid arthritis. WBC = white blood count.***

Of the 3001 people in the RA/methotrexate cohort, 1585 (52.8%) had no abnormalities of any NICE-specified blood tests over 2 years (Table 2). Compared with the whole RA/methotrexate cohort, those with normal tests were generally younger and without comorbidities, particularly chronic kidney disease (CKD) and heart failure (Table 2). Among people aged <60 years, >60% had no test abnormalities compared with <30% among those aged ≥80 years (Table 2). The most common abnormalities were in eGFRs in older people and those with CKD and/or heart failure (Figure 3). Nearly 20% had an abnormal eGFR, which showed a steep age gradient, while 219 (7.3%), 217 (7.2%), and 202 (6.7%) had abnormal ALT, WBC, and neutrophils results, respectively (data not shown). For people with other comorbidities the most common abnormality was also eGFR (Figure 3).

**Table 2.** Characteristics of people with RA and IBD not experiencing blood-test abnormalities over 2 years

**Characteristic**	**People with RA on methotrexate (prevalent and incident) not experiencing any NICE-specified blood-test abnormalities,^a^*n* (%)^b^**	**All people with RA on methotrexate (prevalent and incident), *n* (%)^b^**	**People with IBD on azathioprine (prevalent and incident) not experiencing any of the NICE-specified blood-test abnormalities,^a^ *n* (%)^b^**	**All people with IBD on azathioprine (prevalent and incident), *n* (%)^b^**
**Total**	1585 (52.8)	3001	657 (56.5)	1162

**Age, years**				
Median (range)	63 (53–71)	66 (56–75)	43 (32–54)	44 (32–58)
Mean (SD)	61 (14)	65 (14)	44 (15)	46 (17)

**Age group, years**				
18–39	113 (71.1)	159	279 (57.9)	482
40–59	547 (63.8)	857	275 (65.0)	423
60–79	805 (50.9)	1583	100 (43.5)	230
≥80	119 (29.6)	402	Suppressed^c^	27|

**Sex**				
Male	495 (50.9)	972	330 (58.1)	568
Female	1089 (53.7)	2029	329 (55.4)	594

**Socioeconomic status, IMD quintile**				
1 (most deprived)	208 (52.7)	395	85 (52.5)	162
2	248 (52.9)	469	102 (53.7)	190
3	294 (52.4)	561	131 (54.1)	242
4	355 (53.2)	667	173 (64.1)	270
5 (least deprived)	466 (52.4)	889	157 (56.1)	280
Missing	13 (65.0)	20	11 (61.1)	18

**Long-term conditions at baseline**				
CKD	60 (14.9)	403	10 (21.3)	47
CVD^d^	148 (29.9)	495	16 (23.9)	67
Heart failure	26 (15.5)	168	Suppressed^c^	22
Diabetes	142 (34.0)	418	33 (33.3)	99
Hypertension	524 (41.2)	1271	70 (37.8)	185
Osteoarthritis	429 (39.9)	1076	42 (40.4)	104

a *NICE-recommended test thresholds: white blood count ≥3.5×10^9^/L; mean cell volume ≤105 fL; neutrophils ≥1.6×10^9^/L; platelets ≥140×10^9^/L; eosinophils ≤0.5×10^9^/L; alanine aminotransferase ≤100 U/L; aspartate transaminase ≤100 U/L; albumin ≥30 g/L; and estimated glomerular filtration rate ≥60 ml/min/1.73 m^2^.*

b *Unless otherwise stated.*

c *Data not provided for confidentiality reasons, due to small numbers in this category.*

d *Includes ischaemic heart disease, cerebrovascular disease, and peripheral vascular disease. CKD = chronic kidney disease. CVD = cardiovascular disease. IBD = inflammatory bowel disease. IMD = Index of Multiple Deprivation. NICE = National Institute for Health and Care Excellence. RA = rheumatoid arthritis. SD = standard deviation.*

### IBD/azathioprine

Of the 1162 people in the IBD/azathioprine cohort, 657 (56.5%) had no abnormalities of any NICE-specified blood test conducted in the 2-year study period. The number and proportion of people with test abnormalities were lower overall in the IBD/azathioprine group than in the RA/methotrexate group (Table 2). Again, the pattern of normality/abnormality with age varied between tests, dominated by abnormal renal function in people aged ≥80 years, and those with CKD and heart failure (Figure 3); however, the numbers of older people and those with specific comorbidities were small (Table 2) and, as such, findings should be interpreted cautiously.

WBC was the most common individual abnormality (*n* = 138 people, 11.9%), followed by eGFR (*n* = 88, 7.6%), ALT (*n* = 72, 6.2%), and neutrophils (*n* = 65, 5.6%) (data not shown).

### Multivariable associations

Multivariable regression adjusting for age, sex, socioeconomic status, and long-term conditions showed that, in the RA/methotrexate group, those who were least likely to have normal blood-test results were older and had CKD, heart failure, cardiovascular disease, or diabetes; in the IBD/azathioprine group, those aged 40–59 years were more likely to have normal blood-test results (Table 3).

**Table 3.** Multivariable associations between patient characteristics and not experiencing any blood-test abnormalities^a^ among those with RA who were prescribed methotrexate, and those with IBD who were prescribed azathioprine^b^

**Characteristic**	**Odds ratio**	**95% CI**	***P*-value**
**RA/methotrexate cohort^c^**			

**Age group, years, versus 18–39**			
40–59	0.79	0.55 to 1.13	0.21
60–79	0.56	0.39 to 0.79	<0.001
≥80	0.26	0.17 to 0.40	<0.001

**Sex, versus male**			
Female	1.11	0.94 to 1.30	0.22

**Socioeconomic status, IMD quintile, versus 5 (least deprived)**			
4	0.95	0.77 to 1.17	0.61
3	0.96	0.77 to 1.20	0.70
2	0.95	0.75 to 1.21	0.69
1 (most deprived)	0.90	0.70 to 1.16	0.40

**Long-term conditions**			
CKD versus no CKD	0.37	0.21 to 0.67	<0.001
CVD versus no CVD	0.51	0.30 to 0.85	0.01
Diabetes type 1 or 2 versus no diabetes	0.66	0.51 to 0.83	<0.001
Hypertension versus no hypertension	0.93	0.79 to 1.10	0.41

**IBD/azathioprine cohort^d^**			

**Age group, years, versus 18–39**			
40–59	1.35	1.03 to 1.79	0.03
60–79	0.65	0.45 to 0.93	0.02
≥80	0.09	0.01 to 0.33	0.002

**Sex, versus male**			
Female	0.96	0.75 to 1.22	0.72

**Socioeconomic status, IMD quintile, versus 5 (least deprived)**			
4	1.41	0.99 to 2.01	0.06
3	0.90	0.63 to 1.28	0.55
2	0.83	0.57 to 1.22	0.34
1 (most deprived)	0.85	0.57 to 1.26	0.41

**Long-term conditions**			
CKD versus no CKD	0.39	0.17 to 0.82	0.02
Diabetes type 1 or 2 versus no diabetes	0.57	0.35 to 0.94	0.03
Hypertension versus no hypertension	0.91	0.61 to 1.36	0.64

a *Using NICE-specified thresholds and tests (test thresholds: WBC ≥3.5×10^9^/L; MCV ≤105 fL; neutrophils ≥1.6×10^9^/L; platelets ≥140×10^9^/L; eosinophils ≤0.5×10^9^/L; ALT ≤100 U/L; AST ≤100 U/L; albumin ≥30 g/L; and eGFR ≥60 ml/min/1.73 m^2^).*

b *Models adjusted for age, sex, socioeconomic status, and long-term conditions. Small numbers with CVD and heart failure in the IBD azathioprine group meant this condition combination was not included in the multivariable model as it was for the RA/methotrexate group.*

c *Patients with prevalent and incident RA prescribed methotrexate in any 2-month period over the study period.*

d *Patients with prevalent and incident IBD prescribed azathioprine in any 2-month period over the study period. ALT = alanine aminotransferase. AST = aspartate transaminase. CKD = chronic kidney disease (excluding dialysis and transplant). CVD = cardiovascular disease (including ischaemic heart disease, cerebrovascular disease, heart failure, and peripheral vascular disease). eGFR = estimated glomerular filtration rate. IBD = inflammatory bowel disease. IMD = Index of Multiple Deprivation. MCV = mean cell volume. NSAID = non-steroidal anti-inflammatory drug. RA = rheumatoid arthritis. WBC = white blood cell.*

The small numbers of people in the IBD/azathioprine group aged ≥80 years or having CKD suggests that the precision of the results for these groups should be treated cautiously.

### Absolute risk

In the RA/methotrexate population, absolute risk of persistently normal blood tests was lowest among those aged ≥80 years and those aged 60–79 years with combinations of diabetes, CKD, and cardiovascular disease (see Supplementary Table S3); similar results were found for the IBD/azathioprine group (see Supplementary Table S4 and Supplementary Figure S2).

## DISCUSSION

### Summary

In this large, 2-year retrospective cohort study using linked primary care and laboratory data, the extent of, and factors associated with, persistently normal blood tests were assessed among people with RA taking methotrexate and those with IBD taking azathioprine. It showed that approximately half of those taking methotrexate for RA or azathioprine for IBD experienced no blood-test abnormality using NICE-recommended tests and thresholds. In the RA/methotrexate cohort for whom abnormalities were present, these tended to be in older people with reduced renal function.

For the IBD/azathioprine cohort, individual blood-test abnormalities were less common overall and mainly constituted reduced renal function among a smaller number of older people and those with comorbidities.

The absolute risk of having persistently normal blood tests was lowest among older people and those with comorbidities. A smaller proportion of people with IBD taking azathioprine continuously had regular 3-monthly blood tests, compared with those with RA who were taking methotrexate continuously.

### Strengths and limitations

Strengths of this study included a large sample size that included coding for long-term conditions, linkage to hospital laboratory data (enabling analysis of all blood-test results), and prescribing data at individual level.

There were, however, some limitations. These included: the potential for routine data coding errors to influence the numbers of people coded as having RA and IBD (both prevalent and incident cases) in the dataset; the maximum 2-year follow-up timeframe preventing capture of DMARD-induced abnormalities that might have occurred after this period; and, for those prescribed DMARDs at baseline, a lack of historical prescribing information. If people moved out of the area during the study period their data would stop being captured, meaning that the authors would no longer see information on their blood. There were limited numbers in some subgroups, such as those with IBD and comorbidities, and the proportion of people with RA on methotrexate was also relatively low compared with a previous register-based study.^20^ The latter suggests that there may be people in primary care with coding for RA that reflects a historic diagnosis rather than a currently active problem. However, as the interest of the study presented here was those with both diagnosed RA or IBD *and* prescribed methotrexate or azathioprine, this is unlikely to have had major implications.

It was not possible to consider methotrexate and azathioprine dosage, and some patients may not have been captured if they were mainly cared for in hospital — although this is considered unlikely, as information is shared frequently between primary and secondary care. In addition, there were no data on 6-mercaptopurine, which is used to treat IBD; however, in a recent, large retrospective analysis of data from the IBD BioResource in the UK, of 11 928 participants treated with a thiopurine, the majority (94%) received azathioprine, while around 23% received 6-mercaptopurine (approximately 17% of whom received both).^21^

There was no information on dispensing or adherence (an unavoidable limitation of studying prescribing data) and information was lacking on the reasons for performing blood tests; as such, it was not possible to ascertain which tests were conducted for the purpose of DMARD monitoring. This means that neither higher testing frequency nor blood-test abnormalities can be causally attributed to DMARDs in the study presented here.

The magnitude of abnormalities in relation to the upper limits of normal was not explored. In addition, IBD was not separated into Crohn’s disease and ulcerative colitis, thereby precluding any exploration of differences in prescribing/testing patterns. Finally, as an observational study, there is the potential for unmeasured confounding.

### Comparison with existing literature

This study adds to evidence informing monitoring schedules for DMARDs by suggesting there may be lower-risk populations that are potentially suitable for reduced blood-test monitoring frequencies, particularly younger patients with RA who do not have comorbidities.

Abnormalities were dominated by reduced renal function and, although methotrexate may cause kidney injury directly in rare circumstances, the main purpose of monitoring renal function is the increased risk of methotrexate toxicity, including bone-marrow suppression, in renal impairment and older age.^22^ Cumulative incidence of myelotoxicity was only 7% among 8302 patients with IBD in a meta-analysis of 66 studies — equating to approximately 3% per patient and year of treatment.^23^ The findings from the study presented here do not refute the need for renal-function monitoring, but the high frequency of eGFR testing observed, particularly in patients with RA, suggests there may be some overtesting. In addition, CKD prevalence was 5.2% in the total baseline population, but 13.4% among the RA/methotrexate cohort. Monitoring for myelotoxicity, therefore, remains important, while also remembering that renal function may be reduced but stable. This is useful for GPs considering NICE recommendations for specialist referral.^7^

Low rates of liver-function abnormality were identified, with 7.3% of people with RA on methotrexate and 2.4% of people with IBD on azathioprine having abnormal ALT levels over 2 years. This is in keeping with a systematic review of 47 studies of methotrexate-related liver toxicity in rheumatoid and psoriatic arthritis (mean 3.5 years methotrexate use); the review found a pooled cumulative incidence of elevated liver enzymes of 31%, but showed that these abnormalities were often transient, with methotrexate being continued without adjustment in >65% of patients in 12 of 18 studies.^24^ However, it is important to note that safety considerations may require decisions about optimal blood-testing frequency to be based on risk of more-serious outcomes, rather than transient blood abnormalities. Incidence of severe liver abnormalities in Visser and van der Heijde’s study was low at 4 years of methotrexate use, with severe fibrosis in 1.3% and cirrhosis in 0.5%.^24^ In a large cohort study including 28 030 individuals with RA taking methotrexate, incidence was low at 1.39 and 0.22 per 1000 person–years for mild liver disease and cirrhosis, respectively, during a mean follow-up time of >8 years.^25^ Methotrexate-induced liver abnormalities may appear over long timeframes, which raises concerns about reducing the frequency of testing.^24^^,^^26^^,^^27^

Significant variation in blood-monitoring frequency was found, with most people having ≥9 blood tests over 2 years, illustrating a substantial workload for patients and health services. Blood-test numbers were higher in patients with RA than those with IBD, particularly for eGFR tests, perhaps reflecting older age and higher prevalence of comorbidities. A recent review of blood-test monitoring identified that both under- and overmonitoring are common.^28^ In the study presented here, in the RA/methotrexate and IBD/azathioprine populations prescribed those medicines continually for the whole 2-year period, there were marked differences in undertesting, with <40% of people in the continuous IBD/azathioprine group having tests in every 3-month period, potentially reflecting a higher proportion of the IBD group being of working age.

### Implications for research and practice

Research is needed to identify whether reducing frequency of testing and/or using other tests (such as liver fibrosis markers) would affect the risk of important outcomes, including liver disease. Understanding the acceptability of reducing monitoring among patients and clinicians is also important, as this may cause anxiety for both about the risk of serious and undetected DMARD complications.

Improving the understanding of risk-profile variation among people on DMARDs aligns with UK NHS endeavours towards stratified and personalised medicine, which underline the need to tailor treatments to individual characteristics and adopt more targeted disease-management strategies.^29^^,^^30^ Work is needed to model optimal testing frequencies for people in different risk strata, to consider biological DMARDs, and to reduce unnecessary patient workload. There is a potential to improve monitoring efficiency and sustainability by adjusting monitoring according to risk (if safety implications are considered acceptable).

As populations emerge from the COVID-19 pandemic, there are likely large numbers of people taking DMARDs who have been unable to access blood-test monitoring. A recent survey among European Alliance of Associations for Rheumatology organisations in 35 countries, including the UK, identified that *‘cancellation or postponement of non-urgent tests either by the service provider or by patients themselves were reported by 699/1030 (68%) and 426 (41%) respondents, respectively’*.^31^

The British Society for Rheumatology’s COVID-19 guidelines recommended the need for flexibility in blood testing for patients on stable DMARDs during the pandemic and that cases be reviewed on an individual basis.^32^ The findings presented from the study reported here could, potentially, support prioritisation decisions when catching up with the backlog of people who require testing.

Persistent normality in the results of blood tests used to monitor patients on methotrexate for RA and azathioprine for IBD using NICE-specified thresholds is common, with abnormalities primarily driven by older age and abnormal renal function. More stratified approaches where younger people and those without comorbidities are considered for lower blood-test-monitoring frequency may reduce workload and costs for patients and healthcare providers, particularly in primary care. However, more research is required to determine the long-term safety, acceptability, sustainability, and cost implications of changing monitoring strategies.
